# Prediction of Outcome in Acute Lower Gastrointestinal Bleeding Using Gradient Boosting

**DOI:** 10.1371/journal.pone.0132485

**Published:** 2015-07-14

**Authors:** Lakshmana Ayaru, Petros-Pavlos Ypsilantis, Abigail Nanapragasam, Ryan Chang-Ho Choi, Anish Thillanathan, Lee Min-Ho, Giovanni Montana

**Affiliations:** 1 Department of Gastroenterology, Charing Cross and Hammersmith Hospitals, Imperial College Healthcare NHS Trust, London, United Kingdom; 2 Department of Biomedical Engineering, Kings College London, London, United Kingdom; University Hospital Llandough, UNITED KINGDOM

## Abstract

**Background:**

There are no widely used models in clinical care to predict outcome in acute lower gastro-intestinal bleeding (ALGIB). If available these could help triage patients at presentation to appropriate levels of care/intervention and improve medical resource utilisation. We aimed to apply a state-of-the-art machine learning classifier, gradient boosting (GB), to predict outcome in ALGIB using non-endoscopic measurements as predictors.

**Methods:**

Non-endoscopic variables from patients with ALGIB attending the emergency departments of two teaching hospitals were analysed retrospectively for training/internal validation (n=170) and external validation (n=130) of the GB model. The performance of the GB algorithm in predicting recurrent bleeding, clinical intervention and severe bleeding was compared to a multiple logic regression (MLR) model and two published MLR-based prediction algorithms (BLEED and Strate prediction rule).

**Results:**

The GB algorithm had the best negative predictive values for the chosen outcomes (>88%). On internal validation the accuracy of the GB algorithm for predicting recurrent bleeding, therapeutic intervention and severe bleeding were (88%, 88% and 78% respectively) and superior to the BLEED classification (64%, 68% and 63%), Strate prediction rule (78%, 78%, 67%) and conventional MLR (74%, 74% 62%). On external validation the accuracy was similar to conventional MLR for recurrent bleeding (88% vs. 83%) and therapeutic intervention (91% vs. 87%) but superior for severe bleeding (83% vs. 71%).

**Conclusion:**

The gradient boosting algorithm accurately predicts outcome in patients with acute lower gastrointestinal bleeding and outperforms multiple logistic regression based models. These may be useful for risk stratification of patients on presentation to the emergency department.

## Introduction

Acute lower gastro-intestinal bleeding (ALGIB) is a common emergency increasing in incidence with age [1], and may be more common than acute upper gastrointestinal bleeding in the elderly [2]. The causes and severity are heterogeneous, e.g. large volume bleeding from diverticulosis or minor bleeding from colitis with the aetiology and outcome often obscure to the clinician at presentation.

The majority of ALGIB presentations (80–85%) resolve spontaneously with no adverse outcome and death is uncommon (2–4%) [3,4]. However a significant proportion (17–30%) undergo therapeutic intervention (angiographic embolisation, colonoscopic-based therapies or surgery) to control severe/recurrent bleeding [5,6]. Despite intervention re-bleeding occurs at rate of approximately 20% in the first year [7].

Due to concern about severe/recurrent bleeding or need for intervention routine clinical practice for the vast majority of patients with ALGIB who present to emergency department is admission to hospital for in-patient observation for variable number of days with a proportion undergoing endoscopy or radiological investigation. This strategy has the disadvantage of being invasive, expensive [8] and exposes patients to hospital acquired complications. If a reliable predictive model was available at the point of presentation to hospital low risk patients could be identified and triaged to outpatient management/a shorter inpatient stay. Resources could be then freed up for high-risk patients to be appropriately transferred to higher levels of care and undergo more aggressive investigation. Although multiple logistic regression (MLR) based scores have been developed for ALGIB [9,10] none have been recommended for routine clinical practice unlike the Glasgow Blatchford and Rockall scores for acute upper gastrointestinal bleeding [11]. Possible reasons include the lack of validation in diverse settings and modest accuracies in comparison with these scores. MLR based models may be limited in predicting outcome in ALGIB as they are based on the assumption that a linear combination of the observed features can be used to determine the probability of each particular outcome ignoring any variable interaction which may be the key for the accurate prediction.

In order to mitigate this potential limitation of MLR based scores and to accurately predict ALGIB outcome, we chose to implement and assess the performance of a non-parametric algorithm for classification, Gradient Boosting (GB) [12]. GB is a supervised machine learning algorithm, and is able to approximate the unknown functional mapping between the inputs, i.e. the non-endoscopic measurements, and the outputs, i.e. the ALGIB outcomes. Supervised learning algorithms are commonly trained on historical data consisting of examples of input-output pairs.

The GB algorithm embraces the notion of “ensemble learning”, whereby multiple simple learning algorithms are used jointly in order to obtain better predictive performance than could be achieved from any of the constituent learning algorithms [13]. Of clinical relevance are several reports demonstrating that ensemble learning classification models are accurate in predicting outcome in a variety of clinical settings [14–16].

In particular, the GB algorithm relies on decision trees as constituent or “base” predictive algorithms. Decision tree are statistical models that recursively partition the input space in order to find rules that are predictive of the output. The classical CART (Classification and Regression Tree) algorithm was popularized in the 1980s by Breiman et al. [17]. Compared to other machine learning methods, GB possesses several strengths: 1) it is less prone to over-fitting [13] 2) it is robust to noise [18] 3) it has an internal mechanism to estimate error rates, 4) it provides indices of variable importance and 5) it can be used when the predictors are both continuous and categorical.

The aim of this study was to test whether the GB algorithm was able to accurately predict clinical outcomes in patients presenting to emergency departments with ALGIB using non-endoscopic variables available to clinicians at that time. We also set out to compare the performance of the GB approach with conventional MLR and two previously published multivariate logistic regression models [9,10]. We show in this study that the gradient boosting algorithm accurately predicts rebleeding, severe bleeding and clinical intervention in patients with acute lower gastrointestinal bleeding and outperforms multiple logistic regression based models.

## Methods

### Ethics statement

This study was performed by analysing an existing anonomysed database of patients presenting with ALGIB collated for the purpose of audit to the emergency departments of Charing Cross and Hammersmith hospitals, London, UK (1^st^ January 2007 to 31^st^ December 2011). The study was approved by the Joint Research Compliance office at Imperial College Healthcare NHS Trust (ref 125HH25060). The office confirmed that no formal ethical review or informed consent was required as the study involved existing anonomysed routinely collected data, no new data was being collected and there was no clinical intervention.

### Study design

The database had been generated retrospectively by identifying consecutive patients from electronic records who were aged 18 or over and presented to the emergency department with a primary diagnosis of ALGIB defined as PR bleeding (bright red or maroon coloured blood passed per rectum) within the previous three days. For this study we excluded patients with: 1) presentation most indicative/final diagnosis of an upper GI bleed (haematemesis, melaena, upper GI bleeding source detected at endoscopy or angiography), 2) inpatient ALGIB bleed, 3) ALGIB as a secondary admission symptom, 4) patients transferred from other hospitals and 5) incomplete patient data. Patients with haemodynamic instability and PR bleeding were required to have an upper GI endoscopy before inclusion into the study unless a definitive colonic source was identified.

Patients admitted to Charing Cross Hospital with ALGIB were analysed for training and internal validation of the GB algorithm. The external validation cohort consisted of patients admitted to Hammersmith Hospital over the same time period. Charing Cross and Hammersmith hospitals are large, busy general teaching hospitals with separate emergency departments and surgical teams.

### Definitions of variables and final diagnoses

Data on 39 previously published variables associated with need for intervention or adverse outcome in ALGIB (Table 1) were identified from the literature and collected from prospectively generated databases of presenting diagnoses, laboratory results, endoscopic, discharge and coding databases. The variables were defined as follows: Unstable co-morbidity-any organ system abnormality that usually requires ICU admission, erratic mental status-clouding of consciousness due to any cause or presence of syncope confusion or coma, cardiovascular disease-history of angina, myocardial infarction, cardiomyopathy or heart failure, respiratory disease-current or past history of copd, liver disease-history or presence of jaundice, cirrhosis or portal hypertension and renal failure-creatinine >125mircomol/l.

**Table 1:** Baseline Variables at initial ALGIB presentation.

**Demographic**
Age
Gender
**History**
Use of omeprazole/lansoprazole
Use of NSAID drugs/antocoagulants
Alcoholism
Smoking
Nursing home resident
Colorectal polyp
Haemorrhoids
Diverticular disease history
Colonic AVM
Syncope
**Comorbidities**
Cardiovascular disease
Hypertension
Stroke history
COPD
Chronic renal failure
Diabetes mellitus
Dementia
Cancer
Chronic liver diease
Previous GI bleed history
Unstable comorbidities
**Initial assessment**
Heart rate
Systolic BP
Diastolic BP
Erratic mental status
Abdominal pain
Tender abdominal exam
Ongoing bleed in ED
Gross blood on DRE
**Baseline bloods**
Haemaglobin
Haematocrit
White blood cell count
Platelet
APTT
Prothrombin time
Urea
Creatinine

APTT, activated partial prothrombin time; AVM, arteriovenous malformation; DRE, digital rectal examination; ED emergency department

Definite colonic bleeding was defined as any signs of active or recent bleeding on endoscopy or angiography (stigmata of recent haemorrhage: active bleeding, a non bleeding visible vessel, or an adherent clot). Presumptive was defined haematochezia or blood per rectum and no suspicion of upper GI bleeding, with one or more potential bleeding sources below the ligament of Treitz.

### Gradient boosting

We deployed the GB algorithm, as originally proposed by Friedman [13], which has been successfully applied in a number of clinical applications [19–23]. GB is a non-parametric algorithm for supervised machine learning. It approximates the unknown functional mapping from input explanatory variables to corresponding output variables. The non-parametric nature of the GB algorithm enables the estimation of a functional mapping from non-endoscopic measurements to ALGIB outcomes without the need to decide a priori the parametric form of this function. By contrast, parametric models like logistic regression assume that the log odds depend linearly on the covariates, and this linearity may be insufficient to capture the complexity of relationship between inputs and outputs [24].

For the training of the GB algorithm we consider *m* patients with non-endoscopic input measurements and their corresponding ALGIB outcome in the form of (*x*_1_,*y*_1_),…, (*x*_*m*_,*y*_*m*_) pairs, where each ${\left\{x_{i}\right\}}_{i\;=\;1}^{m}$ is a vector containing the non-endoscopic measurements for patient *i*. We seek to approximate the unknown function *y* = *F*(x*)so that predictions can be made on a new patient for which we have observed the non-endoscopic input measurements x*.

The GB algorithm relies on an iterative model fitting procedure making use of many simple predictive algorithms or “base” learners [13], and combine them to form more complex decision rules. The unknown function *F* is estimated by minimizing a loss function ℒ defined over the training set:

$$F=argmin_{F}\Sigma_{i=1}^{m}L\left(y_{i}\;,\;F\left(x_{i}\right)\right)$$ (1)

GB constructs an approximation *F*_*N*_ of *F* as a sum of *N*+1 “base” learners constructed through N boosting iterations, $F_{N}\;=\;\sum_{n\;=\;0}^{N}f_{n}$. In our implementation, the “base” learners are regression trees ^12^, which are particularly useful in clinical applications as they provide easy to interpret decision rules. GB starts with an initial base learner *F*_0_ and then applies a steepest descent step for the minimization of the loss function with respect to *F*_0_. These two steps are repeated sequentially and each time a new learner is constructed to follow the direction along which the loss of the previous learner is minimized. The steepest descent method takes steps proportional to the negative gradient of the loss function in order to find the local minimum. More explicitly, the gradient of the loss function ℒ for each training point *x*_*i*_ at the iteration step *n* is given by

$$g_{i,n}\left(x_{i}\right)=\nabla_{F_{n-1}\left(x_{i}\right)}L\left(y_{i},F_{n-1}\left(x_{i}\right)\right),\;1\leq \text{i}\leq \text{m.}$$ (2)

The gradient is defined only at the data points ${\left\{x_{i}\right\}}_{i\;=\;1}^{m}$ and cannot be generalized to other *x* values. One way to enable generalization is to choose a regression tree *h*(*x*,*a*_*n*_) that produces $h_{n}\;=\;{\left\{h(x_{i},a_{n})\right\}}_{1}^{m}$ most parallel to-*g*_*n*_ ϵ *ℛ* ^m^. This regression tree can be obtained from the solution

$$a_{n}=argmin_{a,\beta }\;\Sigma_{i=1}^{m}{\left[-g_{n}\left(x_{i}\right)-\;\beta h\left(x_{i},a\right)\right]}^{2},$$ (3)

where *a*_*n*_ are the parameters of the regression tree *h*(*x*,*a*_*n*_) and *β* is the learning rate, which determines the contribution of each tree to the approximation. Having estimated the regression tree that is most highly correlated with-*g*_*n*_ (*x*) over the data distribution, the next update of the approximation *F*_*N*_ is given by

$$F_{n}\left(x\right)=\;F_{n-1}\left(x\right)+\;\gamma_{n}h\left(x,a_{n}\right),$$ (4)

which uses the optimal length,

$$\gamma_{n}=argmin_{\gamma }\;\Sigma_{i=1}^{m}\;L\left(y_{i},\;F_{n-1}\left(x_{i}\right)+\;\gamma h\left(x_{i},a_{n}\right)\right).$$ (5)

The GB algorithm is summarized in the following pseudo-code:

**Gradient Boosting Algorithm**

1 $F_{0}(x)\;=\;{argmin}_{p}\sum_{i\;=\;1}^{m}L(y_{i},p)$, where *p* is the response of the regression tree to the training data.

2 **for** n = 1 → N **do**

3 $\;g_{n}\;=\;\nabla_{F_{n-1}(x)}L(y,F_{n-1}(x))$ gradient at the training data *x*.

4 $\;\;a_{n}\;=\;{argmin}_{a,\beta }\sum_{i\;=\;1}^{m}{\left[-g_{n}(x_{i})-\;\beta h(x_{i},a)\right]}^{2}$, fit a regression tree *h*_*n*_(*x*,*a*_*m*_)

5 *F*_*n*_(*x*) = *F*_n-1_(*x*)+*γ*_*n*_ *h*(*x*,*a*_*n*_), for

6   ${\;\gamma }_{n}\;=\;{argmin}_{\gamma }\sum_{i\;=\;1}^{m}L(y_{i},\;F_{n-1}(x_{i})+\;\gamma h(x_{i},a_{n}))$

7 **end for**

8 **end Algorithm**

After training, the parameters of the learned regression trees enclose rules capturing the (possibly non-linear) relationship between non-endoscopic variables and the ALGIB outcome. A new patient with non-endoscopic measurements x* is then assigned to a specific outcome class *y* simply by following the decision rules associated to that class.

To enable an optimal training of the GB algorithm, first we randomly divided our cohort of 170 patients collected at Charing Cross Hospital into training and validation datasets. Specifically, 70% of patients were assigned to the training set and the remaining 30% were utilized for internal validation. Internal validation was carried out with the objective of optimally tuning the GB hyperparameters (i.e. the number of boosting iterations, the depth of the regression trees and the learning rate) before assessing the performance of the algorithm externally on a cohort of patients admitted to Hammersmith Hospital (completely independent dataset).

For each one of the clinical outcomes in our studies we ranked the covariates using the internal GB mechanism for variable ranking, and selected the 10 best predictive variables for each outcome. We then investigated whether re-fitting the GB algorithm using only this reduced set of covariates would yield comparable performance.

### MLR and published MLR based models for ALGIB

Conventional multiple logistic regression was applied to the Charing Cross and Hammmersmith cohorts using the same 39 non-endoscopic as in GB. Moreover, two published MLR based models for ALGIB, the BLEED score [10] (persistent bleeding, low systolic blood pressure, elevated prothrombin time, erratic mental status and unstable comorbid disease) and Strate prediction rule [9] (heart rate ≥100, systolic blood pressure ≤115, syncope, non tender abdominal examination, rectal bleeding within first 4hrs of evaluation, aspirin use and > 2 co-morbid conditions) were calculated for the both cohorts.

### Outcomes

The outcomes measured were therapeutic intervention (endoscopic, angiographic, surgical), severe bleeding (defined as ongoing or recurrent bleeding) and recurrent bleeding. These outcomes were chosen as they indicate the need for inpatient care. Therapeutic intervention to stop the source of a bleed was included as this suggested the presence of an ongoing bleed that was not resolving spontaneously. Definitions for the three outcomes were taken the published literature [5,9,25,26]. Severe bleeding was defined as the following: continued bleeding in the first 24 hours of hospitalisation (defined as a RBC transfusion of ≥2 units, and/or a haematocrit decrease of ≥20%), or recurrent bleeding after 24 hours of stability (defined as more than one transfusion of RBCs, a further haematocrit decrease of ≥20%, or readmission for ALGIB within 1 week of discharge). Recurrent bleeding was defined as recurrent haematochezia after 24 hours of stabilisation during which no active bleeding was observed, associated with any of the following as a new finding: decrease in haemoglobin of ≥2g/dl, decrease in haematocrit of ≥5%, haemodynamic instability, or having an additional RBC transfusion (≥2 units received in total).

### Statistical Analysis

The following statistical figures to predict severe bleeding, recurrent bleeding and therapeutic intervention were derived for all models in the internal and external validation cohorts: Sensitivity, Specificity, Positive Predictive Value (PPV), Negative Predictive Value (NPV) and Accuracy (sum of correct predictions over total predictions) using HDS Epimax, 2004 and Graph Pad Prism software. Comparison of continuous and categorical data (between the internal and external validation cohorts) was carried out using Mann-Whitney U and Fisher exact tests respectively. A two-tailed significance of 5% was used in all comparisons.

## Results

### Characteristics and clinical outcomes of cohorts

For the Charing Cross cohort (CXC) following the initial search through emergency and endoscopy databases at Charing Cross hospital 174 patients were identified as having had a history of ALGIB and presentation to the emergency department. Four patients were excluded due to incomplete information for risk scoring. The 170 remaining patients made up the training/internal validation cohort. The same search process was used to compile the Hammersmith cohort (HC). 133 patients were attended the emergency department of Hammersmith hospital with a primary diagnosis of ALGIB. Of these patients three were excluded due incomplete data for risk scoring. The remaining 130 patients made up the external validation cohort. The accuracy of data collection was by random re-assessment of 10% of notes by another author (LA).

The demographic characteristic, clinical features and final diagnoses of the internal and external validation cohorts are shown in Tables 2 and 3. The patients in the internal Charing Cross cohort were similar to the Hammersmith cohort with regard to sex ratio, median age and length of stay. Nearly all patients (98–100%) in both cohorts were admitted to hospital for management of ALGIB which lasted a median of 4 days. Upper GI endoscopy to rule out an upper GI bleed was carried out in 20% of cases in the internal cohort and 14% of cases in the external cohort. Patients in both cohorts were similar in terms of undergoing a lower endoscopy procedure (74% vs. 81%) but in-patient colonoscopy was more common in the Charing Cross cohort (76% vs. 46%). This consisted of colonoscopy (56% Charing cross cohort, 45% Hammersmith cohort) with the remainder having flexible sigmoidoscopy, rigid sigmoidoscopy or proctoscopy. A CT or mesenteric angiogram was carried out in 8% of patients in the Charing Cross cohort and 5% in the Hammersmith cohort.

**Table 2:** Characteristics and outcomes of cohorts.

Characteristic	Charing Cross (n = 170)	Hammersmith (n = 130)	P value
Male	90 (53%)	69 (53%)	ns
Median Age (range)	70 (16–99)	70 (17–101)	ns
Discharge within 24 hrs	4 (2%)	0 (0%)	ns
Median, mean hospital stay (range)	4, 7 (0–102)	4, 7 (0–182)	ns
Blood cell transfusion	58 (34%)	23 (18%)	0.001
Had lower GI Endoscopy	125 (74%)	105 (81%)	ns
**Outcomes**			
Severe bleeding	60 (35%)	28 (22%)	0.01
Recurrent bleeding	34 (20%)	19 (14%)	ns
Therapeutic intervention	26 (15%)	9 (7%)	0.02
endoscopic	9 (5%)	4 (3%)	ns
angiographic	9 (5%)	4 (3%)	ns
surgery	8 (4.7%)	1 (0.7%)	ns
Death	4 (2.3%)	3 (2.3%)	ns

ns-not significant

**Table 3:** Final Diagnoses in Cohorts.

	Charing Cross (n = 170)	Hammersmith (n = 130)
**Definite**		
Diverticulosis and its complications	13	16
Colitis	14	5
Anorectal (including varices)	6	12
Neoplasia and post-neoplasia therapy	14	5
Angiodysplasia	3	4
Isolated large bowel ulcers	3	1
Coagulation disorders	1	0
Small bowel bleeding	1	1
**Presumptive**		
Diverticulosis and its complications*	44	13
Colitis	11	11
Anorectal (including varices)	11	9
Neoplasia and post-neoplasia therapy	2	4
Angiodysplasia	3	2
Coagulation disorders	0	1
solated large bowel ulcers	4	0
Small bowel bleed	1	0
No diagnosis made*	39	46

* significant difference between cohorts p<0.05

The three most common diagnoses were diverticulosis, colitis and anorectal disorders such as haemorrhoids. Final diagnoses were similar in both cohorts apart from a presumptive diagnosis of a diverticular bleed which was more common in the Charing Cross cohort and no diagnosis made which was more common in the Hammersmith cohort.

Therapeutic intervention for bleeding (endoscopic therapy, angiographic embolisation or surgery) was more common in the Charing Cross cohort. Endoscopic intervention in both cohorts consisted of clipping, APC and banding. Angiographic embolisation of colonic vessels was carried out in nine patients in the internal cohort and four in the external cohort. Blood transfusion was significantly more common in the Charing Cross cohort (mean 1.6 units vs. 0.9 units) as was severe bleeding. There was no significant difference between the cohorts in re-bleeding or death. All patients who died were ≥65 years of age and the causes of death were cancer (n = 2) cardiac failure (n = 1), pneumonia (n = 1), colonic ischemia (n = 1), pulmonary hypertension (n = 1) and unknown (n = 1). No patient died because of uncontrolled bleeding.

### Predictive performance of gradient boosting and multiple logistic regression

The best GB algorithms using the 39 variables had predictive accuracies of 88%, 91% and 83% (Table 4) for recurrent bleeding, therapeutic intervention and severe bleeding respectively. The accuracies were similar in the Charing Cross and Hammersmith cohorts. The positive predictive value of all GB algorithms were not high in either cohort although importantly for clinical decision making the negative predictive values were high (88–98%) for the three outcomes. The top ten contributing predictors used in the GB algorithms for each outcome are listed on Table 5 in descending order. Four variables (heart rate, diastolic blood pressure, creatinine and APTT) were in the top ten most frequently used variables for all outcomes. On internal validation the accuracy of the GB models for predicting recurrent bleeding, therapeutic intervention and severe bleeding was (88%, 88% and 78% respectively) and superior to the BLEED classification (64%, 68% and 63%) (Table 4), Strate prediction rule (78, 78, 67%) and conventional MLR (74%, 74% 62%). On external validation the accuracy of the GB algorithm was similar to conventional MLR for recurrent bleeding (88% vs. 83%), and therapeutic intervention (91% vs. 87%) but superior for severe bleeding (83 vs 71%). GB models using just the top ten predictive variables were less accurate in predicting rebleeding, severe bleeding and therapeutic intervention than those with the full set of 39 variables by 8–10% on average.

**Table 4:** Predictive performance of models in Charing Cross (CXC) and Hammersmith (HC) cohorts.

Outcome variable	Accuracy	Sensitivity	Specificity	PPV	NPV
**GB model: Recurrent bleeding**					
CXC	88	67	91	50	95
HC	88	57	91	50	94
**GB model: Therapeutic intervention**					
CXH	88	80	89	44	98
HC	91	60	92	27	98
**GB model: Severe bleeding**					
CXH	78	73	80	61	88
HC	83	57	89	58	90
**MLR model: Recurrent bleeding**					
CXC	74	22	85	25	83
HC	83	20	85	6	95
**MLR model: Therapeutic intervention**					
CXC	74	16	82	11	97
HC	87	20	90	9	96
**MLR model: Severe Bleeding**					
CXC	62	46	69	39	75
HC	71	35	83	42	80
**BLEED classification (CXC only)**					
Recurrent bleeding	64	24	75	21	77
Therapeutic intervention	68	27.5	76	19	84
Severe bleeding	63	33	79	44	69
**Strate prediction rule (CXC only)cut off >3**					
Recurrent bleeding	78	16	94	33	81
Therapeutic intervention	78	4	92	8	84
Severe bleeding	67	13	96	66	66

**Table 5:** Top ten variable importance using gradient boosting models.

	Contribution %
**Severe bleeding**	
Platelet count	13.4
APTT	13.0
Haematocrit	12.4
Urea	10.9
Creatinine	9.7
Prothrombin time	8.9
Diastolic blood pressure	6.8
Heart rate	4.1
Systolic blood pressure	3.9
Alcohol abuse	3.9
**Therapeutic Intervention**	
Haemoglobin	15.7
Diastolic blood pressure	13.9
haematocrit	9.5
APTT	9.0
Creatinine	8.2
Fresh blood on PR	7.1
Prothrombin time	6.7
Heart rate	5.0
Past medical history of colorectal polyp	3.4
Use of NSAIDs or anticoagulants	3.4
**Recurrent bleeding**	
Creatinine	19.1
Haemoglobin	18.8
Age	17.9
Diabetes	13.2
APTT	11.5
Diastolic blood pressure	6.8
Heart Rate	4.6
Urea	4.4
Alcoholism	2.4
Total number of co-morbidities	1.3

## Discussion

There is a need for non-endoscopic risk scores to help risk stratify patients with ALGIB for early discharge/outpatient management or higher levels of care and thereby utilise resources efficiently. Current clinical practice includes colonoscopic-based triage which is invasive, adequate preparation difficult to achieve and treatable stigmata of haemorrhage infrequent [1]. This study has shown that a GB algorithm based on clinical and laboratory variables was accurate (>80%) in predicting the clinical outcomes of recurrent bleeding, therapeutic intervention and severe bleeding.

GB had high negative predictive values (88–98%) in both Charing Cross and Hammersmith Cohorts. This suggests that these models may be useful to triage patients into a low-risk group who could be managed with an abbreviated stay in hospital avoiding high levels of care or as outpatients. The median and mean inpatient stay in the Charing Cross and Hammersmith cohorts was four and seven days respectively and therefore a reduction in this would allow for significant cost-savings and decrease exposure of patient to hospital associated hazards such as infections.

A particular strength of this study is the validation and good performance in an external cohort with a lower incidence of severe bleeding indicating the algorithm can maintain accuracy in a different setting. Our GB algorithm used only non-endoscopic variables available to the clinician in the emergency department and therefore has clinical applicability for decision-making. We would however emphasise that such a model is not aimed at replacing experienced decision making but rather aiding the process. This is the case with recommended risk scores for acute upper gastrointestinal bleeding such as the Rockall and Glasgow Blatchford scores [11].

One study to our knowledge has examined an ensemble machine learning model in acute gastrointestinal bleeding and was developed to identify the bleeding source, need for resuscitation and those who require urgent endoscopy [27]. This study differs substantially from ours in that a random forests algorithm was used by building classification trees ignoring the error of the previous tree in the sequence [28]. Also a mixed population of patients with both acute upper, middle and lower gastrointestinal bleeding was studied (no numbers given for ALGIB), there was no comparison with previously published scores and the model was not tested in an external cohort. Nevertheless accuracies of >75–80% were found for the studied outcomes providing evidence of the utility of the ensemble machine learning techniques.

The performance of the GB algorithm in our study was superior to MLR and two published MLR based models. Ensemble machine learning models have been shown to be more accurate than conventional logistic regression to classify disease or predict outcome in a variety of clinical settings [29–31]. Theoretical reasons for this are that logistic regression predicts outcomes based on linear combinations of independent variables by fitting a single model that best explain the relationship between observed values and outcome. On the other hand, the rationale of the GB algorithm to fit many simple models whose predictions are then combined can produce a good fit of the predicted outcome values to the observed values, even if the specific nature of the relationship between the predictor variables and the corresponding outcome is complex (e.g. nonlinear, interacted or noisy with outliers). Also, unlike multiple logistic regression, GB method can handle a large number of input variables and generate an internal unbiased estimate of the generalization error as the simple classification tree estimation progresses. Finally, the stage-wise model fitting procedure of the GB algorithm allows to automatically assess the influence of each non-endoscopic variable in the construction of a robust classification rule [12].

Other explanations are that the BLEED score [10] was designed to predict a composite endpoint of in-hospital complication (recurrent haemorrhage, surgery to control haemorrhage and hospital mortality) rather than the end-points we examined. The Strate prediction rule [9] was designed to predict severe bleeding which we studied but in our cohort performed least well for this outcome and better for recurrent bleeding and need for clinical intervention. Artificial neural networks, another machine learning classifier, have also been shown to be accurate in predicting re-bleeding and clinical intervention in ALGIB [5]. However for our cohort, the classification performance of neural networks was found inferior to GB in ALGIB (data not shown).

Our study has a number of potential limitations: First the database of patients was collated retrospectively and relied on the inherent accuracy of patient records. The majority of data collected was however quantitative and also available from prospectively generated electronic laboratory, endoscopic and patient records. Second the GB requires the input of many more variables than BLEED or Strate prediction rule (39 vs. 5 vs. 8) which increases complexity. Reduction in the number of variables used in the GB model to 10 led to decreased accuracy of 8–10% for the studied outcomes which would compromise clinical utility of the GB algorithm. Importantly however in our experience input of data for the 39 variables into the programme takes less than 5 minutes and therefore would be suitable for use in an emergency department/ward particularly given the explosion of smart phone apps which allow for quick data entry with drop down menus where only positive inputs are required. This would be similar to endoscopy reports which are generated electronically, typically require >50 pieces of data and are used in routine clinical practice. Third the decision to give blood transfusion and apply endoscopic therapy was not protocol based which could have led to bias. In mitigation our analysis showed that endoscopic therapy was consistently applied in this study according to consensus guidelines (data not shown) and therefore limits this as a potential source of bias. Finally death was not examined as an outcome due its infrequent nature in our cohort. Death is rare in ALGIB occurring in <4% in large series and generally occurs in those with co-morbid conditions [6] and after an in-patient bleed [32] that latter which was an exclusion criteria for our study. Future work to examine the GB model in a larger cohort could be undertaken to examine its utility in predicting death. To determine the impact of the model in predicting outcomes in ALGIB a randomised study of the GB model plus routine clinical decision-making versus routine clinical decision-making could be performed.

In summary, gradient boosting accurately predicts outcome in patients with acute lower gastrointestinal bleeding. This machine learning approach has the potential to aid in the risk stratification of patients with ALGIB on presentation to the emergency department.
